# Co-ultramicronized Palmitoylethanolamide/Luteolin Promotes the Maturation of Oligodendrocyte Precursor Cells

**DOI:** 10.1038/srep16676

**Published:** 2015-11-18

**Authors:** Massimo Barbierato, Laura Facci, Carla Marinelli, Morena Zusso, Carla Argentini, Stephen D. Skaper, Pietro Giusti

**Affiliations:** 1 Department of Pharmaceutical and Pharmacological Sciences, University of Padua, 35131 Padua, Italy

## Abstract

Oligodendrocytes have limited ability to repair the damage to themselves or to other nerve cells, as seen in demyelinating diseases like multiple sclerosis. An important strategy may be to replace the lost oligodendrocytes and/or promote the maturation of undifferentiated oligodendrocyte precursor cells (OPCs). Recent studies show that a composite of co-ultramicronized N-palmitoylethanolamine (PEA) and luteolin (co-ultramicronized PEA/luteolin, 10:1 by mass) is efficacious in improving outcome in experimental models of spinal cord and traumatic brain injuries. Here, we examined the ability of co-ultramicronized PEA/luteolin to promote progression of OPCs into a more differentiated phenotype. OPCs derived from newborn rat cortex were placed in culture and treated the following day with 10 μM co-ultramicronized PEA/luteolin. Cells were collected 1, 4 and 8 days later and analyzed for expression of myelin basic protein (MBP). qPCR and Western blot analyses revealed a time-dependent increase in expression of both mRNA for MBP and MBP content, along with an increased expression of genes involved in lipid biogenesis. Ultramicronized PEA or luteolin, either singly or in simple combination, were ineffective. Further, co-ultramicronized PEA/luteolin promoted morphological development of OPCs and total protein content without affecting proliferation. Co-ultramicronized PEA/luteolin may represent a novel pharmacological strategy to promote OPC maturation.

Oligodendrocytes are the myelin-producing cells of the central nervous system (CNS)^1^. Myelin, a lipid-rich membrane, insulates the axons of neurons thereby allowing the rapid conduction of electrical impulses and delivery of the action potential to the target cell^2^,^3^. Loss of myelin leads to a range of neurological disorders, including reduced motor function, impaired cognitive abilities, and vision problems. Among demyelinating diseases affecting the CNS, multiple sclerosis (MS) has probably received the most attention. MS typically strikes young adults (with a higher incidence in women), and is the most common cause of chronic neurological impairment in young people^4^. Lesions in CNS white and gray matter, identifiable by magnetic resonance imaging, are characteristic of MS patients^5^,^6^,^7^. Further, MS lesions are distinguished by the presence of undifferentiated oligodendrocyte precursor cells (OPCs), highlighting their inability to mature into myelin-producing oligodendrocytes^8^. Inflammation in these lesions is caused by an immune response to myelin^9^,^10^. Although widely believed to be immune-mediated and pathologically attributable to myelin-specific autoreactive CD4^+^ T cells, the humoral autoimmune response in MS is probably not restricted to myelin but is much more widespread throughout the brain. The complex heterogeneity of MS is suggested by the finding that autoantibodies are formed against different CNS cell types, including neurons, oligodendrocytes, astrocytes, and immune cells^11^.

Different therapeutic strategies are available for treatment of MS including immunosuppressants, immunomodulators, and monoclonal antibodies^12^,^13^. Intended to target the recurring inflammation of the disease, they do not necessarily ensure remyelination. Indeed, considerable efforts are now being directed to the next phase of MS therapy, namely, remyelination/regeneration^14^,^15^,^16^,^17^. The antimuscarinic antiparkinsonian agent benztropine has been reported to stimulate OPC differentiation *in vitro* and promote remyelination in mouse models of MS^14^. However, potential dose-dependent side-effects are associated with benztropine treatment in man^18^.

It has been proposed that chronic neuroinflammation is sustained by an imbalance between pro-inflammatory and pro-resolving lipid mediators, thereby inhibiting a physiological program of resolution and promoting the progression of persistent neuroinflammation^19^. Some investigators^20^ have further suggested that these lipid mediators might be leveraged to induce a *“dominant program of resolution.*” The N-acylethanolamines represent one such family of lipid signalling molecules, produced ‘on demand’ by tissue damage or an inflammatory response. Among these, N-palmitoylethanolamine (palmitoylethanolamide, PEA) has been extensively studied in experimental models of acute and chronic inflammatory pain, neuropathic pain, cerebral ischemia, traumatic brain and spinal cord injury, and neurodegenerative diseases^21^,^22^,^23^. Moreover, the preclinical literature has been validated through clinical trials of PEA mainly in the area of chronic and neuropathic pain^24^. Recent studies, moreover, suggest that a co-ultramicronized composite of PEA and the flavonoid luteolin (‘co-ultramicronized PEA/luteolin ‘), when compared to either molecule alone or in simple combination, exerts superior anti-inflammatory action while improving neurological outcome in experimental models of spinal cord injury^25^, traumatic brain injury^26^, and Alzheimer disease^27^. Based on these intriguing findings, the experiments described in this report were carried out to investigate the possibility of co-ultramicronized PEA/luteolin to effect the maturation of cortical OPCs *in vitro*.

## Results

### Co-ultramicronized PEA/luteolin promotes the morphological development of OPCs

**Figure 1:**
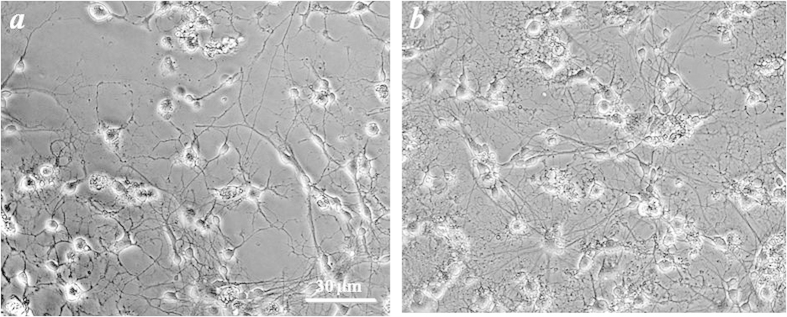
Co-ultramicronized PEA/luteolin promotes the morphological development of cortical oligodendrocyte precursor cells. One day after plating OPCs were treated with 10 μM co-ultramicronized PEA/luteolin as detailed in Methods. Following a further 4 days of incubation the cultures were photographed under phase contrast microscopy. Note what appears to be a more complex morphology and greater extent of branching in cells treated with co-ultramicronized PEA/luteolin (**b**) compared to untreated cells (**a**). Scale bar: 30 μm.

**Figure 2:**
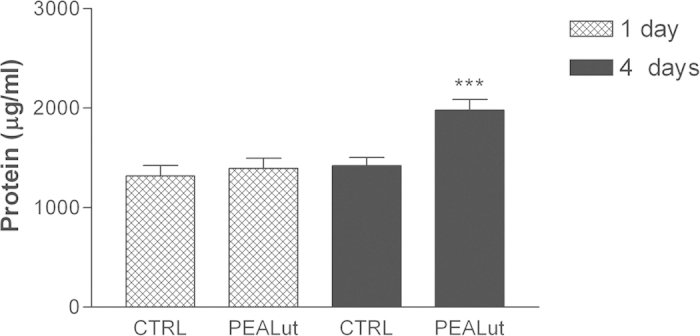
Co-ultramicronized PEA/luteolin treatment increases the total protein content of differentiating OPCs. Cultures of OPCs were treated the day after plating with 10 μM co-ultramicronized PEA/luteolin (‘PEALut’) as detailed in Methods. Cultures were harvested 1 day and 4 days later and protein content measured (expressed as μg/ml cell lysate). Data are means ± s.e.m. (n = 4–8). ***p < 0.001 *vs* control at 4 days (CTRL, 0.02% Pluronic F-68).

Treatment of cultured OPCs with 10 μM co-ultramicronized PEA/luteolin, starting the first day after plating resulted in a increase in complexity and abundance of ramifications typical of non-myelinating mature oligodendrocytes when observed four days later (Fig. 1)^1^. In addition, cultures were processed for myelin basic protein (MBP) and proteolipid protein (PLP) immunocytochemistry after 1 and 4 days. MBP and PLP are the two major structural myelin proteins of the CNS^1^,^28^,^29^, and are expressed by non-myelinating and myelinating mature oligodendrocytes^1^,^30^. Cultures after 1 day were immunonegative for both myelin proteins but positive after 4 days in both control and treated, with a clear overlap in immunostaining for MBP and PLP (Supplementary Fig. S1). Cells treated with co-ultramicronized PEA/luteolin appeared to display a greater extent of branching at the later time (evidenced by a halo of puncta), reminiscent of non-myelinating mature oligodendrocytes^1^,^30^,^31^. A detailed immunocytochemical characterization of these cultures has been described in detail earlier (see ref. 32 in Methods). Further, the total protein content of the OPC cultures increased modestly, but significantly, over this time (Fig. 2). Neither co-ultramicronized PEA/luteolin at 1 μM, nor the single components were efficacious (data not shown). While this increase in protein content could result from OPC proliferation, treatment with co-ultramicronized PEA/luteolin (10 μM) failed to promote expression of Ki-67 mRNA (Supplementary Table S1), a well-accepted proliferation marker^33^,^34^. Conceivably, this effect might also reflect an increased tissue mass or cell hypertrophy of the cultures.

### Co-ultramicronized PEA/luteolin regulates mRNA expression of myelin genes in differentiating OPCs

**Figure 3:**
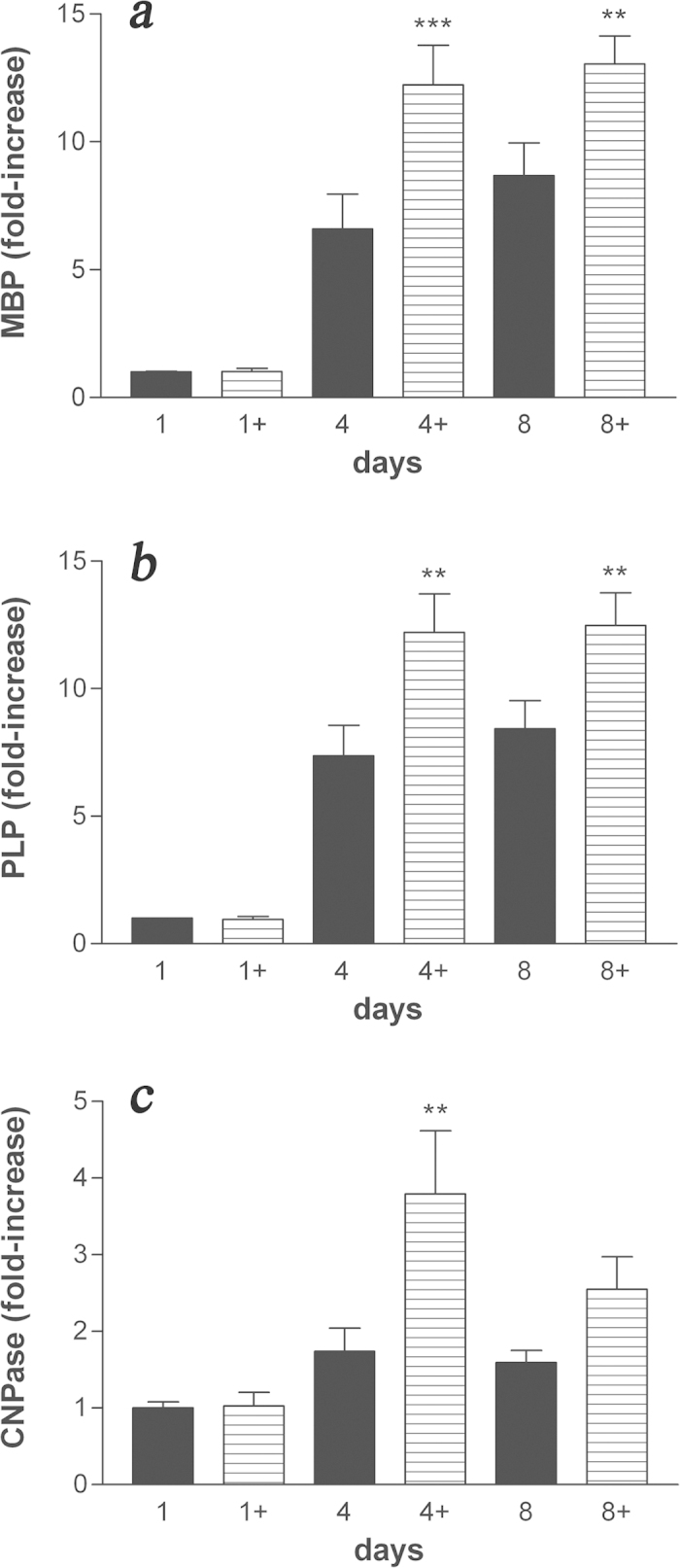
Treatment of differentiating OPCs with co-ultramicronized PEA/luteolin up-regulates, in a time-dependent manner mRNA for: (**a**) myelin basic protein (MBP); (**b**) proteolipid protein (PLP); (**c**) 2′,3′-cyclic nucleotide 3′-phosphodiesterase (CNPase). Cultures of OPCs were treated the day after plating with 10 μM co-ultramicronized PEA/luteolin (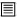) (indicated by ‘+’) and processed 1, 4 and 8 days later for RT-PCR, as detailed in Methods. Data are expressed as fold-increase with respect to the control (vehicle only) at 1 day (set to 1) and are means ± s.e.m. (panel *a*, n = 4–9; panel *b*, n = 4–6; panel *c*, n = 5 for 1 and 4 days, n = 3 for 8 days). **p < 0.01 *vs* vehicle (■) (0.02% Pluronic F-68); ***p < 0.001 *vs* vehicle.

**Figure 4:**
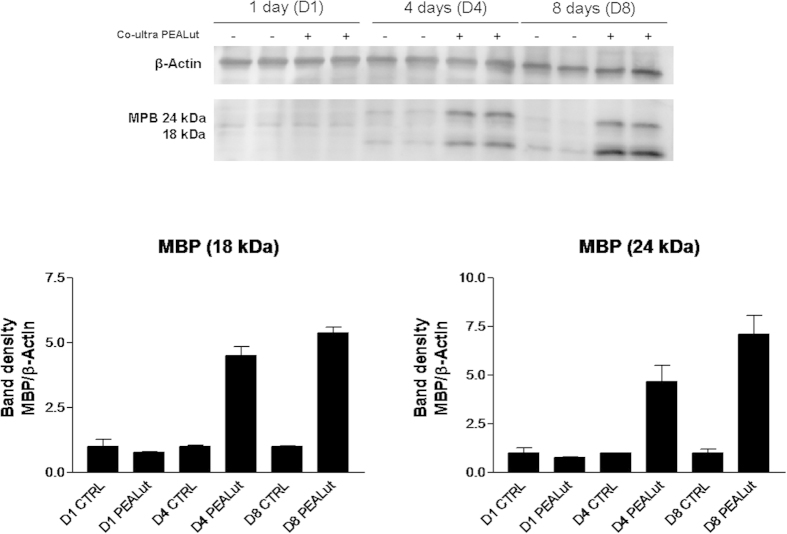
Co-ultramicronized PEA/luteolin stimulates expression of myelin basic protein (MBP) 18 kDa and 24 kDa isoforms in differentiating OPCs. Cultures of OPCs were treated the day after plating with 10 μM co-ultramicronized PEA/luteolin (‘PEALut’) and cell lysates prepared 1 (D1), 4 (D4) and 8 (D8) days later for Western blot analysis, as detailed in Methods. Data are expressed relative to amount of MBP in untreated cells for each respective time point (=1) and are means for duplicate samples. Similar results were obtained in two other experiments.

Based on these initial observations, we next examined the ability of co-ultramicronized PEA/luteolin to induce the expression of markers characteristic of a more mature oligodendrocyte phenotype. Incubation of OPCs with co-ultramicronized PEA/luteolin (10 μM), commencing the day after cell plating led to a time-dependent rise in the expression of MBP and PLP mRNA levels by quantitative PCR, which was significantly greater compared to vehicle-treated cells (Fig. 3a,b). The effect of co-ultramicronized PEA/luteolin was concentration-dependent, increasing MBP gene expression after 4 days by 0.90 ± 0.08, 1.37 ± 0.22 and 1.85 ± 0.02-fold at 0.1, 1 and 10 μM, respectively (mean ± s.e.m., n = 3, p < 0.001 vs vehicle at 10 μM). This was accompanied by a corresponding increase in the cellular content of both the 18 kDa and 24 kDa isoforms of MBP up to 8 days (Fig. 4). The membrane-anchored myelin enzyme 2′,3′-cyclic nucleotide 3′-phosphodiesterase (CNPase)^1^, which is believed to mediate process outgrowth in oligodendrocytes^35^ and play a critical role in the events leading up to myelination^36^ also displayed elevated gene levels in OPCs treated with co-ultramicronized PEA/luteolin over this time, as compared to vehicle alone (Fig. 3c). Coincidentally with this up-regulated expression of myelin protein genes, platelet-derived growth factor receptor alpha (PDGFRα), a recognized marker of OPCs but not premyelinating or myelinating oligodendrocytes^1^,^31^, fell by about 95% by day 4 and remained so until day 8, and was not influenced by incubation with co-ultramicronized PEA/luteolin at any time point (Supplementary Fig. S2). Interestingly, the magnitude of this drop in PDGFRα expression matches the percentage of differentiating immunocyochemically characterized oligodendrocytes in these cultures, based on our earlier study (see Methods, ref. 32).

**Figure 5:**
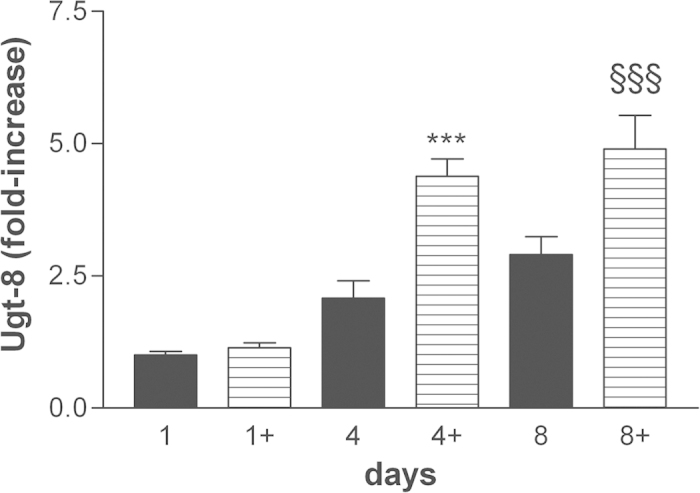
UDP glycosyltransferase 8 (Ugt-8) mRNA levels are up-regulated by co-ultramicronized PEA/luteolin in differentiating OPCs. Cultures of OPCs were treated the day after plating with 10 μM co-ultramicronized PEA/luteolin (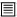) (indicated by ‘+’) and processed 1, 4 and 8 days later for RT-PCR, as detailed in Methods. Data are expressed as fold-increase with respect to the control (vehicle only) at 1 day (set to 1) and are means ± s.e.m. (n = 5 for 1 and 4 days, n = 3 for 8 days). ***p < 0.001 *vs* vehicle (■) (0.02% Pluronic F-68); ^**§§§**^*p* < 0.001 *vs* vehicle.

**Figure 6:**
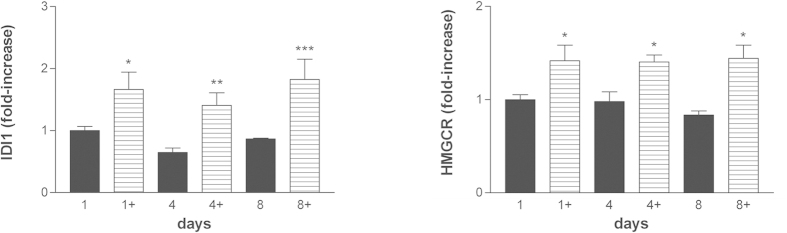
Co-ultramicronized PEA/luteolin regulates cholesterol synthesis genes in differentiating OPCs. Cultures of OPCs were treated the day after plating with 10 μM co-ultramicronized PEA/luteolin (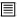) (indicated by ‘+’) and processed 1, 4 and 8 days later for RT-PCR, as detailed in Methods. (*left panel*): isopentenyl-diphosphate delta isomerase 1, IDI1; (*right panel*): HMG-CoA reductase, HMGCR. Data are expressed as fold-increase with respect to the control (vehicle only) at 1 day (set to 1) and are means ± s.e.m. (n = 5 for 1 and 4 days, n = 3 for 8 days). *p < 0.05 *vs* vehicle (■) (0.02% Pluronic F-68); **p < 0.01 *vs* vehicle; ***p < 0.001 *vs* vehicle.

**Figure 7:**
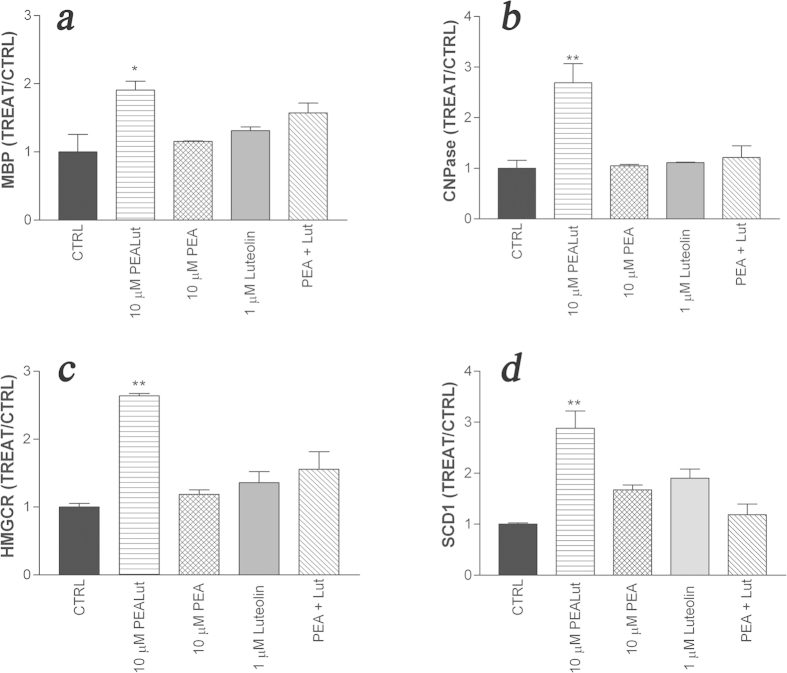
Co-ultramicronized PEA/luteolin regulation of gene expression at day 6 in differentiating OPCs is not mimicked by its molecular components alone. Cultures of OPCs were treated the day after plating (day 2) with: 10 μM co-ultramicronized PEA/luteolin (‘PEALut’), 10 μM ultramicronized PEA (‘PEA’), 1 μM luteolin, or 10 μM ultramicronized PEA + 1 μM luteolin (‘PEA+Lut’). Cells were harvested 4 days later (day 6) and processed for RT-PCR, as detailed in Methods. (**a**) myelin basic protein (MBP); (**b**) 2′,3′-cyclic nucleotide 3′-phosphodiesterase (CNPase); (**c**) HMG-CoA reductase (HMGCR); (**d**) stearoyl.CoA desaturase-1(SCD1). Data are given with reference to vehicle (CTRL, 0.02% Pluronic F-68), set to 1. Values are means ± s.e.m. (n = 3). *p < 0.05 *vs* vehicle; **p < 0.01 *vs* vehicle.

Myelin is also dependent on its characteristic lipid contents, which provide critical building blocks of this membrane structure^37^. Therefore, it is important to assess as well the potential regulatory effects of co-ultramicronized PEA/luteolin on lipogenesis. We measured the mRNA levels of UDP glycosyltransferase 8 (Ugt-8), which catalyzes the final step in the biosynthesis of cerebrosides^38^, a major component of myelin membranes up-regulated during oligodendrocyte differentiation^39^. Here also, OPCs incubated with co-ultramicronized PEA/luteolin (10 μM) showed a time-dependent increase in gene expression in comparison to vehicle-only cultures (Fig. 5). In addition, genes involved in cholesterol synthesis (HMG-CoA reductase, HMGCR; isopentenyl-diphosphate delta isomerase 1, IDI1) and fatty acid synthesis (stearoyl CoA desaturase-1, SCD1) were analyzed. As Fig. 6 shows, co-ultramicronized PEA/luteolin (10 μM) up-regulated mRNA expression of IDI1 (left panel) and HMGCR (right panel) in differentiating OPCs already after 1 day of treatment, and remained above control through 8 days – in contradistinction to the more delayed rise in the genes for MBP and PLP. Co-ultramicronized PEA/luteolin significantly up-regulated also mRNA levels for SCD1 when examined after 4 days of treatment (Fig. 7d). A number of other genes (Ki-67, Supplementary Table S1; PDGFRα, Supplementary Fig. S2; cannabinoid receptor 2 and cannabinoid receptor 1, Supplementary Table S1; superoxide dismutase 2 (SOD2), Supplementary Fig. S3) were unaltered, suggesting that co-ultramicronized PEA/luteolin effects may not reflect a general induction of transcription.

The effects of co-ultramicronized PEA/luteolin on OPC development were not mimicked by either ultramicronized PEA (10 μM), luteolin (1 μM), or the combined treatment with ultramicronized PEA (10 μM) plus luteolin (1 μM). This was the case for expression of genes involving both myelin proteins and pathway components of lipogenesis. Figure 7 describes several examples for illustrative purposes, for cells analysed after 4 days of treatment. The effective concentrations of co-ultramicronized PEA/luteolin in the present study, and the superior efficacy of the co-ultramicronized composite in comparison to its constituent molecules are consistent with prior reports^25^,^26^,^27^. Increasing the luteolin concentration to 10 μM was found, following a 4-day incubation to compromise cell vitality. Ultramicronized PEA alone, up to 20 μM was without effect; above this concentration one encounters potential vehicle issues and solution behaviour.

### mTOR and the OPC-differentiating action of co-ultramicronized PEA/luteolin

**Figure 8:**
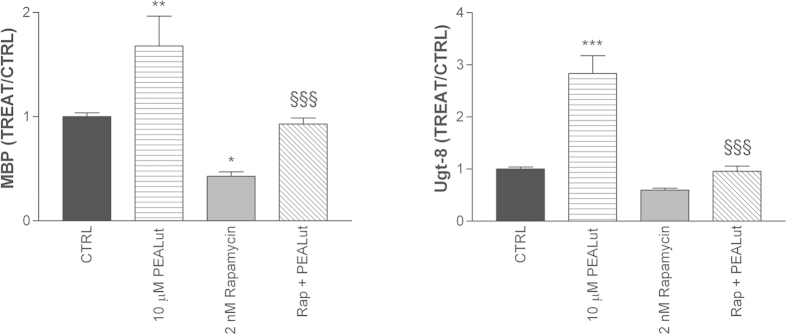
mTOR inhibitor rapamycin blocks the co-ultramicronized PEA/luteolin up-regulation of mRNA for myelin basic protein (MBP) and UDP glycosyltransferase 8 (Ugt-8) in differentiating OPCs. Cultures of OPCs were pre-treated 30 min the day after plating with 2 nM rapamycin (‘Rap’), followed by addition of co-ultramicronized PEA/luteolin (‘PEALut’, 10 μM final) and processed 4 days later for RT-PCR, as detailed in Methods. (*left panel*): MBP; (*right panel*): Ugt-8. Data are expressed relative to the mRNA level in vehicle-treated (0.02% Pluronic F-68) control cells (‘CTRL’) (=1) and are means ± s.e.m. (n = 11–14 for MBP, n = 6–18 for Ugt-8). *p < 0.05 *vs* CTRL; **p < 0.01 *vs* CTRL; ^**§§§**^*p* < 0.001 *vs* co-ultramicronized PEA/luteolin. The rapamycin + co-ultramicronized PEA/luteolin group did not differ significantly from the control group.

**Figure 9:**
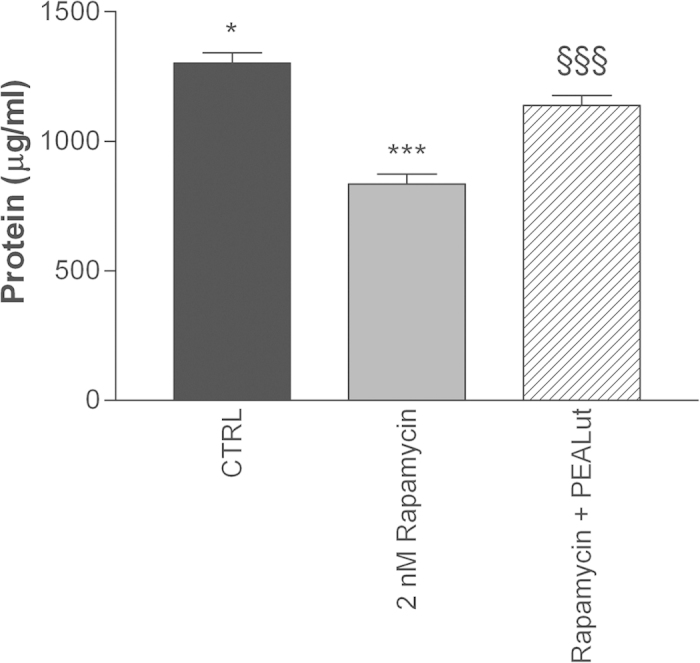
mTOR inhibitor rapamycin reduces the protein content in cultures of differentiating OPCs. Cultures of OPCs were pre-treated 30 min the day after plating with 2 nM rapamycin, followed by addition of co-ultramicronized PEA/luteolin (‘PEALut’, 10 μM final) or not. Cultures were harvested 4 days later and protein content measured (expressed as μg/ml cell lysate). Data are means ± s.e.m. (n = 6). *p < 0.05 for rapamycin + co-ultramicronized PEA/luteolin *vs* CTRL; ***p < 0.001 for rapamycin *vs* vehicle (CTRL, 0.02% Pluronic F-68); ^§§§^*p* < 0.001for rapamycin *vs* rapamycin + co-ultramicronized PEA/luteolin.

Emerging evidence now points to mammalian target of rapamycin (mTOR) as playing a key role in oligodendrocyte differentiation and myelin^40^,^41^,^42^,^43^,^44^,^45^. Rapamycin, an established, selective inhibitor of mTOR was used to interrogate a role for this kinase in OPC differentiation. When added the day after cell plating, 2 nM rapamycin (effective concentrations as described by others^40^,^45^) reduced the expression levels of mRNA for MBP and Ugt-8 in differentiating OPCs when analysed 4 days later (Fig. 8, left and right panels, respectively). Co-ultramicronized PEA/luteolin appeared able to partially restore, at least to control levels, MBP and Ugt-8 gene transcripts. Analogous results were obtained for PLP gene expression (data not shown). Further, OPC cultures treated with 2 nM rapamycin displayed an attenuated morphological maturation and lower protein content after 4 days, which co-ultramicronized PEA/luteolin (10 μM) was able to overcome, in part (Fig. 9).

## Discussion

Oligodendrocytes, the myelin-producing cells of the CNS, are the main target in chronic immunological diseases such as MS, the latter’s pathophysiological pattern characterized by inflammatory cell infiltration, demyelination, axonal damage, astrogliosis, and neurodegenerative processes^4^. Currently, approved disease-modifying therapies are based on the use of immunomodulatory agents, several of which can be administered orally^46^. However, they do not improve disease outcome after degeneration occurs, and therefore are insufficient to treat chronic neurological disability in patients with progressive MS. A promising perspective for future disease therapy is the regeneration of lesions with replacement of the damaged oligodendrocytes^14^,^15^,^16^,^17^,^47^,^48^. As one step in this direction, we identify a novel property of co-ultramicronized PEA/luteolin in enhancing the maturation of differentiating cortical OPCs *in vitro* by stimulating the expression of myelin-specific genes (MBP, CNPase, and PLP) as well as cellular content of MBP. In addition, of co-ultramicronized PEA/luteolin increased the expression of genes involved in lipogenesis, including Ugt-8 which catalyzes the final step in the biosynthesis of cerebrosides^38^.

The fatty acid amide signalling molecule PEA has been shown in a number of studies to display anti-neuroinflammatory and neuroprotective actions^21^,^22^,^23^. Following these reports, more recent investigations indicate that a composite containing PEA co-ultramicronized with the flavonoid luteolin may possess an enhanced pharmacological profile in these experimental models^25^,^26^,^27^,^49^. Flavonoids like luteolin are polyphenolic phytochemicals with potent antioxidant capacity and exhibit neuroprotective/anti-inflammatory actions^50^. Among the family of flavonoids, luteolin is claimed to possess memory-improving^51^ and anxiolytic^52^ effects, and not wholly explained by its antioxidant capacity. Treatment of differentiating OPCs with co-ultramicronized PEA/luteolin resulted in a time-dependent increase in mRNA levels for the major myelin proteins MBP and PLP, together with the genes for constituent enzymes involved in lipid biosynthesis. The timing of gene expression was neither synchronous nor sustained in all cases: for example, IDI1 and HMGCR mRNAs were already elevated after 1 day of incubation with co-ultraPEA/Lut, and remained at that level between 4 and 8 days; Ugt-8 mRNA was first increased at 4 days, and remained significantly elevated at 8 days of co-ultramicronized PEA/Lut treatment. This is perhaps not surprising, as the differentiation of OPCs is a rather complex process requiring the coordinated expression of genes to achieve maturation. Importantly, the rise in MBP mRNA was accompanied by a marked and *sustained* increase in the cellular content of MBP protein. These effects of co-ultramicronized PEA/luteolin are not necessarily the result of a general induction of transcription, as other genes (e.g. PDGFRα, Ki-67, cannabinoid receptors 1 and 2, SOD2) were unaltered. These observations with the composite of PEA and luteolin, and ineffectiveness of its constituent molecules are consistent with other studies^25^,^26^,^27^,^49^. The mechanistic basis underlying the actions of co-ultramicronized PEA/luteolin remains to be investigated. PEA actions are known to be pleiotropic in nature and involve interaction with a number of targets, including peroxisome proliferator-activated receptor alpha, the transient receptor potential vanilloid type 1 receptor and GPR55^22^.

Oligodendrocytes have the highest rate of oxidative metabolic activity in the brain, a likely consequence of the energy consumption needed for biosynthesis of lipid-rich myelin^53^. This may result in the generation of large amounts of reactive oxygen species, the latter playing a role in inflammation and inflammatory demyelinating disorders, such as MS and experimental autoimmune encephalomyelitis^54^,^55^. As oligodendrocytes are the brain’s predominant iron-containing cells, this fact may also contribute to their susceptibility to oxidative injury – coupled with low glutathione levels^56^,^57^. In particular, one major contributor to oxidative damage is H_2_O_2_, which is converted from superoxide that leaks from the mitochondria. Catalase and superoxide dismutase ameliorate the damaging effects of H_2_O_2_ and superoxide, respectively, by converting these compounds into oxygen and H_2_O_2_ (which is later converted to water)^58^. Interestingly, incubation of oligodendrocytes at day 6 in culture with 10 μM co-ultramicronized PEA/luteolin for 24 h produced a two-fold increase in catalase mRNA level (Supplementary Fig. S3). Co-ultramicronized PEA/luteolin at a lower concentration (1 μM) was ineffective; neither concentration altered expression of SOD2 (Supplementary Fig. S3).

mTOR is a serine/threonine protein kinase that controls cell growth, proliferation and survival. A number of studies support a role for mTOR signalling in oligodendrocyte differentiation and myelination^40^,^41^,^42^,^43^,^44^,^45^. Rapamycin (also known as sirolimus), a potent and selective inhibitor of mTOR, regulated the expression of myelin genes in differentiating OPCs (Fig. 8), consistent with previous reports^40^. In addition, rapamycin blunted the ability of co-ultramicronized PEA/luteolin to promote the biochemical maturation of the differentiating OPCs, suggesting that co-ultramicronized PEA/luteolin may act via a mTOR-dependent molecular pathway. Further studies will be needed to elucidate this possibility.

Activated microglia and macrophages play a key role in driving demyelination during MS^59^. Mast cells, also a component of the innate immune system, are undoubtedly major contributors to autoimmune disease, as well^60^. Given the likelihood that microglia, mast cells and oligodendrocytes interact with each other^61^,^62^, approaching MS as both an inflammatory and neurodegenerative disease has important implications for treatment, with remyelination of axons to protect neurons from damage being necessary in addition to controlling inflammation^63^. The findings reported here encourage the view that co-ultramicronized PEA/luteolin may represent a potentially novel avenue in the treatment of inflammatory demyelinating disorders, and merits further investigation in appropriate animal models as a next step. Of consequence, neither PEA^24^ not luteolin^64^, at pharmacologically relevant doses, have been reported to show adverse effects.

## Methods

### Materials

Tissue culture media, N2 supplement, antibiotics and fetal calf serum (FCS) and NP40 cell lysis buffer (10×) were obtained from Life Technologies (San Giuliano Milanese, Italy); poly-D-lysine hydrobromide (mol wt 70,000–150,000), poly-L-lysine hydrobromide (mol wt 70,000–150,000), cytosine β-D-arabinoside, 3,3′,5-triiodo-L-thyronine, L-thyroxine, rapamycin, luteolin, papain, DNase I (bovine pancreas), trypsin inhibitor, protease inhibitor cocktail, Pefabloc® SC (100 mM), Pluronic F68 and all other biochemicals were purchased from Sigma-Aldrich (Milan, Italy) unless noted otherwise; Falcon tissue culture plasticware was purchased from BD Biosciences (SACCO srl, Cadorago (CO), Italy). Sterilin petri plastic dishes (10 cm Ø) were from Sarstedt (Verona, Italy). Co-ultramicronized PEA/luteolin (10:1 mass ratio) and ultramicronized PEA were kindly provided by Epitech S.p.A., Saccolongo (PD), Italy.

### Primary culture of oligodendrocyte precursors

Experiments were performed in accordance with the National Institutes of Health guidelines for the care and use of laboratory animals and those of the Italian Ministry of Health (D.L. 116/92). The University of Padua Institutional Animal Care and Use Committee approved the experimental protocols used in this study. Mixed glial cell cultures from cortex were prepared from postnatal day 1 rat pups (strain: CD) as previously described^32^,^65^. Briefly, tissue dissociates were plated in 75-cm^2^ poly-L-lysine-coated (10 μg/ml) tissue culture flasks at a density of 1.5 brains and grown in high-glucose Dulbecco’s modified Eagle’s medium with 2 mM glutamine, 100 units/ml penicillin/50 μg/ml streptomycin, 50 μg/ml gentamicin and 10% FCS. Medium was changed after 24 h. Upon reaching confluence (approximately 7-8 days later) microglia adhering to the astroglial monolayer were dislodged by shaking the flask for 1 h at 200 rpm in a rotatory shaker (37 °C). This medium was discarded and the flasks re-fed with fresh medium and returned to the incubator for another 2 days. These flasks were subjected to a second cycle of rotary shaking (6 h); the culture supernatant was subsequently transferred to plastic Petri dishes (Sterilin) and incubated for 30 min at 37 °C (5% CO_2_/95% air) to allow differential adhesion of any remaining microglia. The final cell suspension (characterized previously as greater than 93% A2B5 negative and positive for O1 antigen, CNPase and galactocerebroside^32^) was collected and centrifuged (200*g*, 5 min). The resulting cell pellet was re-suspended in Sato’s medium (high-glucose Dulbecco’s modified Eagle’s medium supplemented to contain 400 ng/mL 3,3′,5-triiodo-L-thyronine, 400 ng/ml L-thyroxine, 2 mM L-glutamine, 50 U/ml penicillin and 50 μg/ml streptomycin, 5 ml N2 supplement) and 0.5% (v/v) FCS. Cortical OPCs were seeded in poly-D-lysine-coated (10 μg/ml) 24-well plates at a density of 450,000 cells per well, or in 96 well microplates (72,000 cells per well) in Sato’s medium and maintained at 37 °C in a 5% CO_2_/95% air incubator. After 24 h cytosine β-D-arabinoside (10 μM; to inhibit growth of any residual astrocytes) was added.

### Preparation of co-ultramicronized PEA/luteolin solutions

Co-ultramicronized PEA/luteolin was prepared as a 5 mM stock solution in 10% (w/v) Pluronic F-68. Concentration was calculated based on the molecular weight of PEA (the co-ultramicronized PEA/luteolin composite contains PEA and luteolin in a 10:1 mass ratio). The co-ultramicronized PEA/luteolin solution was sonicated for 20 min in a Elmasonic S (Singen, Germany) sonicating water bath. The co-ultramicronized PEA/luteolin solution was then diluted into Sato’s medium at 100x the desired final concentration, and added (10 μl/1 ml) directly to the cell cultures without exchange of medium. Stock solutions (5 mM) of ultramicronized PEA and luteolin were prepared/sonicated in the same manner; from these the co-respective working solutions were prepared. The concentration of Pluronic F-68 was maintained constant at 0.02%, and added to the control culture wells also. In experiments where ultramicronized PEA and luteolin were added together the final concentration of Pluronic F-68 was 0.022%; this had no effect of cell behaviours. In those experiments where rapamycin was introduced (Figs 8 and 9), the compound was first prepared as a 20 μM stock solution in dimethylsulfoxide (DMSO) and then diluted 100-fold in Sato’s medium (0.2 μM working solution, 1% DMSO). The latter was added to the cultures (10 μl/1 ml) to give a final concentration of 2 nM rapamycin (0.01% DMSO final). After 30 min incubation co-ultramicronized PEA/luteolin was added, as above. All cultures (including control) contained a final concentration of 0.02% Pluronic F-68 and 0.01% DMSO.

### Real Time-Polymerase Chain Reaction (RT-PCR)

The day following plating, OPCs were incubated with: co-ultramicronized PEA/luteolin (1-10 μM final concentration), 10 μM ultramicronized PEA, 1 μM luteolin, or 10 μM ultramicronized PEA and 1 μM luteolin added individually. At various times (as indicated in the corresponding figure legend) total RNA was extracted from cells by TRIzol (Invitrogen), according to the manufacturer’s instructions. RNA integrity and quantity were determined by RNA 6000 Nano assay in an Agilent BioAnalyser. RT was performed with Superscript III reverse transcriptase (Invitrogen). The RT-PCR reaction was performed as described previously^66^. Primer sequences are listed in Table 1. Amounts of each gene product were calculated using linear regression analysis from standard curves, demonstrating amplification efficiencies ranging from 90 to 100%. Dissociation curves were generated for each primer pair, showing single product amplification. Data are normalized to β-actin mRNA level.

### Immunocytochemistry

OPCs were cultured on poly-D-lysine-coated 8-well chamber slides (Labtek). After allowing 1 h for cells to attach co-ultramicronized PEA/luteolin was added to a final concentration of 10 μM. Following 1 day and 4 days of incubation the cells were fixed with 4% paraformaldehyde for 30 min at 4 °C, and washed 4 × 5 min with phosphate-buffered saline (PBS)/0.05% Triton X-100, and blocked with PBS/10% FCS for 1 h at room temperature. The cells were then processed for immunostaining with primary antibodies against MBP (mouse monoclonal, Santa Cruz Biotechnology, Heidelberg, Germany, 1:400), or PLP (Abcam, rabbit polyclonal, 1:200). Cells were then washed 5 × 5 min with PBS, and incubated for 1 h at room temperature with goat anti-rabbit AlexaFluor555 (red) or goat anti-mouse AlexaFluor488 (green) secondary antibody (1:500, Invitrogen). Chamber slides were mounted beneath glass slides using Fluoromount-G (Southern Biotech, USA), and images were acquired on a Leica DMI4000 B microscope equipped for immunofluorescence (Leica Microsystems GmbH, Wetzlar, Germany) using a Leica DFC 480 digital camera.

### Western blot

The day following plating, OPCs were treated with: co-ultramicronized PEA/luteolin (1–10 μM final concentration), 10 μM ultramicronized PEA, 1 μM luteolin, or 10 μM ultramicronized PEA and 1 μM luteolin added individually in combination. At various times (as indicated in the corresponding figure legend) cell lysates were prepared as follows: wash cell monolayers with cold phosphate-buffered saline and add per well 40 μl of lysis solution (890 μl NP40 cell lysis buffer (Life Technologies, 100 μl protease inhibitor cocktail (Sigma-Aldrich), 10 μl of 0.1 M Pefabloc SC (Fluka)), leave on ice for 30 min, collect extracts and clear by centrifugation at 13,000 rpm for 10 min (Microfuge^®^ 22R centrifuge, Beckman Coulter). Supernatants were stored at −80 °C. Protein content in lysates was quantified using the BCA Protein Assay Reagent Kit (Pierce) according to the manufacturer’s protocol. Protein samples (10 μg) were separated on a Mini-PROTEAN^®^ TGX Precast Gel (Bio-Rad, Milan, Italy) with a 4–15% gradient for 70 min at 100V. Proteins were electrophoretically transferred from the gel onto polyvinylidene difluoride (Merck Millipore, Milan, Italy) membranes 90 min at 100V. Membranes were blocked with 3% bovine serum albumin (Sigma-Aldrich) for 1 h at room temperature and then incubated overnight at 4 °C with one of the following primary antibodies: MBP (Santa Cruz Biotechnology, Heidelberg, Germany, 1:200); β-actin (Sigma-Aldrich, 1:25000). The membranes were washed and then incubated for 1 h with the appropriate secondary antibody (goat anti-rabbit (Bio-Rad) or goat anti-mouse (Millipore) conjugated to horseradish peroxidase at a dilution of 1:3000. Development was performed using an enhanced chemiluminescence substrate (Sigma-Aldrich). Immunreactivity was visualized using the VersaDoc Imaging System (Bio-Rad) and protein expression normalized to β-actin for band density quantification.

### Statistics

Data are given as mean ± s.e.m. Statistical analyses to determine group differences were performed either by two-sample equal variance Student’s *t*-test, or by one-way analysis of variance, followed by Dunnett’s or Bonferroni’s post-hoc tests for comparisons involving more than two data groups. Significance was taken at p < 0.05.

## Additional Information

**How to cite this article**: Barbierato, M. *et al.* Co-ultramicronized Palmitoylethanolamide/Luteolin Promotes the Maturation of Oligodendrocyte Precursor Cells. *Sci. Rep.* **5**, 16676; doi: 10.1038/srep16676 (2015).

## Supplementary Material

Supplementary Information

## Figures and Tables

**Table 1:** List of PCR primers

Target	Primer	
Beta actin (Actb)	Forward	5′-GATCAGCAAGCAGGAGTACGATGA-3'
	Reverse	5′-GGTGTAAAACGCAGCTCAGTAACA-3'
Cannabinoid receptor 1 (Cnr1)	Forward	5′-GCATGATTCAGCGTGGGACC-3′
	Reverse	5′-GCCAGCCTAATGTCCATGCG-3′
Cannabinoid receptor 2 (Cnr2)	Forward	5′-TCCTGGCCAGCGTGATCTTT-3'
	Reverse	5′-GAAGGTCATGGTCACGCTGC-3'
Catalase (Cat)	Forward	5′-AAACCCGATGTCCTGACCAC-3'
	Reverse	5′-CATCTCGTCGGTGAAAACCA-3'
2′,3′-cyclic nucleotide 3′ phosphodiesterase (Cnp)	Forward	5′-GGATGAACCCAAGGAGAAGCT-3'
	Reverse	5′-ATTTGGTTGTACAGTGCAGCA-3'
3-hydroxy-3-methylglutaryl-CoA reductase (Hmgcr)	Forward	5′-GGGGCGTGCAAAGACAATCC-3'
	Reverse	5′-TCAAGGACAACTCACCAGCCA-3'
Isopentenyl-diphosphate delta isomerase 1 (Idi1)	Forward	5′-GTTGTTTCACCAATAGTTGCTGT-3'
	Reverse	5′-GCCTCTGTGCTGCTCGTTTG-3'
Mki67	Forward	5′-AGTGCCTTGCTCCAGGTGAA-3'
	Reverse	5′-GCAAGTCTGTTTGGCCACTGT-3'
Myelin basic protein (Mbp)	Forward	5′-TCCGAGGAGAGTGTGGGTTT-3'
	Reverse	5′-TGGAACGATCTGGAGGGTTT-3'
Proteolipid protein 1 (Plp1)	Forward	5′-CCCTGACTGTTGTATGGCTCCT-3'
	Reverse	5′-GCAATAGACTGGCAGGTGGT-3'
Stearoyl-Coenzyme A desaturase 1 (Scd1)	Forward	5′-GATCAAGGCAGGCAGGGAGT-3'
	Reverse	5′-CCCGAAGTGAGGTCCTGAGC-3'
Superoxide dismutase 2 (Sod2)	Forward	5′-CATTGTGCCTCTGGGTTTTT-3'
	Reverse	5′-GCCCTGCATACTTTGTCCAT-3'
UDP glycosyltransferase 8 (Ugt8)	Forward	5′-CTCCATCAGCCCAACTCGGT-3'
	Reverse	5′-GTGACTCGTCTCCCTGTTCCA-3'
Platelet-derived growth factor receptor alpha (PDGFR-α)	Forward	5′-CGTCAACATCAGCGCCTCAC-3′
	Reverse	5′-AGCCAGTTCACGCCCACATA-3′
