# Supplementary material for: Co-ultramicronized Palmitoylethanolamide/Luteolin Promotes the Maturation of Oligodendrocyte Precursor Cells
Source: Sci Rep. 2015 Nov 18;5:16676. doi: 10.1038/srep16676 (PMC4649338; doi:10.1038/srep16676)
Supplement: Supplementary Information [file srep16676-s1.pdf]

Supplementary Information

Co-ultramicronized Palmitoylethanolamide / Luteolin Promotes the Maturation of Oligodendrocyte Precursor Cells

Massimo Barbierato, Laura Facci, Carla Marinelli, Morena Zusso, Carla Argentini, Stephen D. Skaper and Pietro Giusti

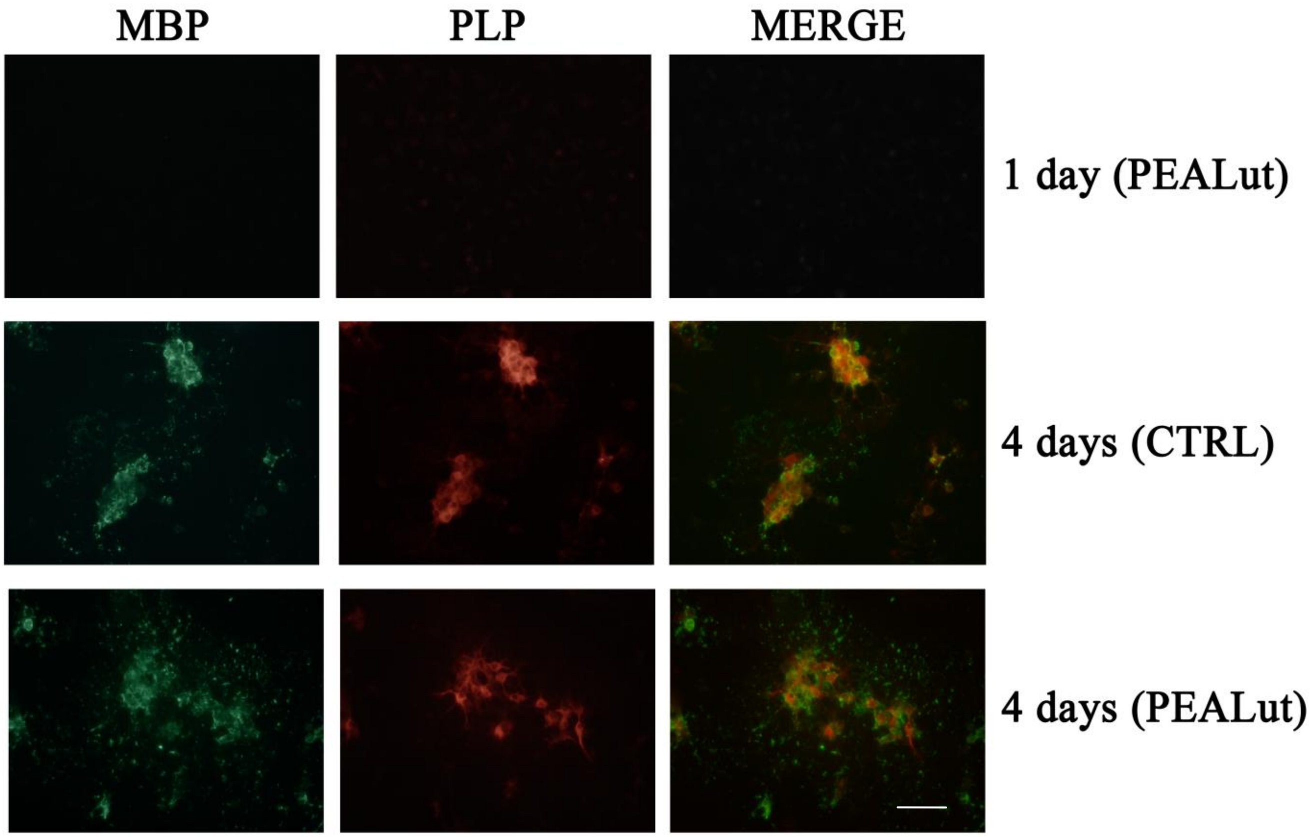

Supplementary Figure S1. Co-ultramicronized PEA/luteolin promotes the morphological development of cortical oligodendrocyte precursor cells: immunocytochemical analysis. One day after plating OPCs were treated with 10 μM co-ultramicronized PEA/luteolin as detailed in Methods. Following a further 1 day and 4 days of incubation the cultures were paraformaldehyde-fixed and processed for myelin basic protein (MBP) and proteolipid protein (PLP) double immunocytochemistry as described in Methods. Note that no immunoreactivity for either protein was observed after 1 day, while both MBP (green) and PLP (red) immunostaining was seen after 4 days. The far-right column shows MBP and PLP co-reactivity in the cells at the later time point. Cells treated with co-ultramicronized PEA/luteolin appeared to display a greater extent of branching (note halo of puncta). Scale bar: 30 µm.

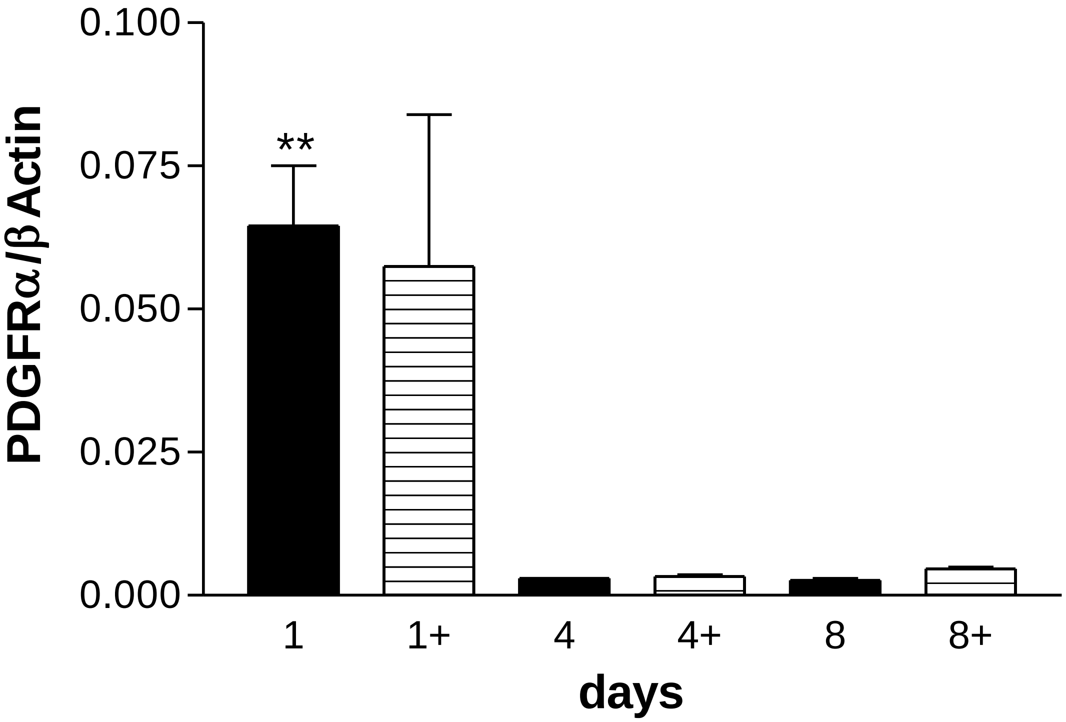

Supplementary Figure S2. Platelet-derived growth factor receptor alpha (PDGFRα) gene expression diminishes with time in culture in differentiating OPCs. In this experiment parallel groups of cultures were treated with 10 μM co-ultramicronized PEA/luteolin (indicated by hatched bars and '+'); untreated cultures (0.02% Pluronic F-68 only) are indicated by solid bars. Cultures were harvested 1 day, 4 days and 8 days later and processed for RT-PCR, as detailed in Methods. Data are means ± s.e.m. (n=3). ***p*<0.01 *vs* vehicle at day 4 and day 8. Co-ultramicronized PEA/luteolin did not alter PDGFRα mRNA expression at any time point. Similar results were obtained in a second experiment. Note the drastic decrease (~95%) in PDGFRα mRNA levels as the OPCs develop.

Supplementary Table S1. Co-ultramicronized PEA/luteolin does not stimulate gene expression for the proliferation marker Ki-67 or cannabinoid receptors CB2 and CB1 in differentiating OPCs.

**Treatment**	**Treated/Control**		
	Ki67	***CB2 receptor***	***CB1 receptor***
Control	1.000 ± 0.062	1.000 ± 0.025	1.000 ± 0.091
PEALut	1.025 ± 0.092	0.947 ± 0.057	1.019 ± 0.136

Cultures of OPCs were treated the day after plating with 10 μM co-ultramicronized PEA/luteolin ('PEALut'), as detailed in Methods. Cells were harvested 4 days later and processed for RT-PCR. Data are given with reference to vehicle (0.02% Pluronic F-68). Values are means ± s.e.m. (n=4).

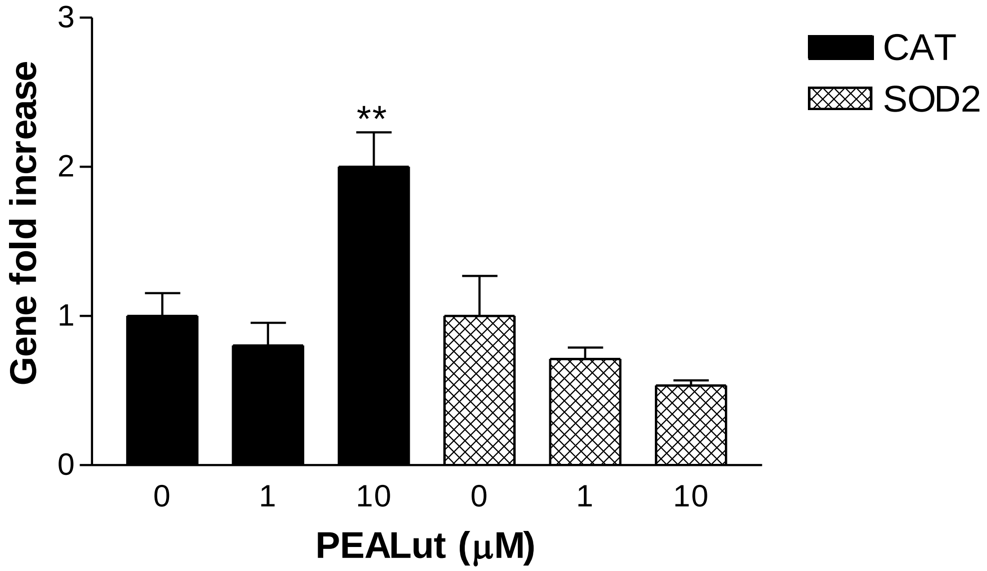

Supplementary Figure S3. Effect of co-ultramicronized PEA/luteolin on catalase (CAT) and superoxide dismutase 2 (SOD2) mRNA expression in cortical oligodendrocytes. Cultures at day 6 were incubated for 24 h with 1 and 10 μM co-ultramicronized PEA/luteolin ('PEALut') and then processed for mRNA analysis by quantitative PCR, as detailed in Methods. Data are given with reference to vehicle (0.02% Pluronic F-68), set to 1, and are means ± s.e.m. (n=3). ***p*<0.01 *vs* vehicle. Similar results were obtained in a second experiment.
